# Recommended strategies for spectral processing and post-processing of 1D ^1^H-NMR data of biofluids with a particular focus on urine

**DOI:** 10.1007/s11306-018-1321-4

**Published:** 2018-02-12

**Authors:** Abdul-Hamid Emwas, Edoardo Saccenti, Xin Gao, Ryan T. McKay, Vitor A. P. Martins dos Santos, Raja Roy, David S. Wishart

**Affiliations:** 1 Imaging and Characterization Core Lab, KAUST, Thuwal, 23955-6900 Kingdom of Saudi Arabia; 2 Laboratory of Systems and Synthetic Biology, Wageningen University and Research, Stippeneng 4, 6708 WE Wageningen, The Netherlands; 3 Computer, Electrical and Mathematical Sciences and Engineering Division, Computational Bioscience Research Center, King Abdullah University of Science and Technology (KAUST), Thuwal, 23955 Kingdom of Saudi Arabia; 4 Department of Chemistry, University of Alberta, Edmonton, Canada; 5 Centre of Biomedical Research, Formerly, Centre of Biomedical Magnetic Resonance, Sanjay Gandhi Post-Graduate Institute of Medical Sciences Campus, Lucknow, India; 6 Department of Biological Sciences, University of Alberta, Edmonton, Canada

**Keywords:** Spectral processing, NMR spectroscopy, Metabolomics, Data post-processing, Baseline correction, Spectral alignment, Spectral binning, Normalization, Scaling, Urine

## Abstract

^1^H NMR spectra from urine can yield information-rich data sets that offer important insights into many biological and biochemical phenomena. However, the quality and utility of these insights can be profoundly affected by how the NMR spectra are processed and interpreted. For instance, if the NMR spectra are incorrectly referenced or inconsistently aligned, the identification of many compounds will be incorrect. If the NMR spectra are mis-phased or if the baseline correction is flawed, the estimated concentrations of many compounds will be systematically biased. Furthermore, because NMR permits the measurement of concentrations spanning up to five orders of magnitude, several problems can arise with data analysis. For instance, signals originating from the most abundant metabolites may prove to be the least biologically relevant while signals arising from the least abundant metabolites may prove to be the most important but hardest to accurately and precisely measure. As a result, a number of data processing techniques such as scaling, transformation and normalization are often required to address these issues. Therefore, proper processing of NMR data is a critical step to correctly extract useful information in any NMR-based metabolomic study. In this review we highlight the significance, advantages and disadvantages of different NMR spectral processing steps that are common to most NMR-based metabolomic studies of urine. These include: chemical shift referencing, phase and baseline correction, spectral alignment, spectral binning, scaling and normalization. We also provide a set of recommendations for best practices regarding spectral and data processing for NMR-based metabolomic studies of biofluids, with a particular focus on urine.

## Introduction

NMR has played an important role in the development and the continuing advances in metabolomics over the past two decades. Indeed, the very first metabolomics papers were based on NMR spectral analysis of biofluids, such as urine (Serkova et al. 2005; Bertram et al. 2006; Gibney et al. 2005; Beckonert et al. 2007b; Bales et al. 1986). Even today there are more than 600 papers published each year that describe the use of NMR in metabolomics studies. Continuing improvements in NMR technology, such as increased magnet field strength (> 1 GHz) (Cousin et al. 2016; Tkac et al. 2009; Abdul-Hamid M.; Emwas et al. 2013), cryogenically cooled probe technology (Keun et al. 2002), microprobe design advances (Miao et al. 2015; Nagato et al. 2015; Grimes and O’Connell 2011) and dynamic nuclear polarization (Emwas et al. 2008; Ludwig et al. 2010) have significantly improved the sensitivity of NMR for metabolomics applications. Now samples as small as 50 µL are being handled and nanomolar concentrations are now detectable. Despite not being quite as sensitive as MS-based metabolomics (Grison et al. 2016; Zhao et al. 2016; Emwas and Kharbatia 2015; Emwas 2015), NMR spectroscopy has several advantages. In particular, NMR requires: (1) little sample preparation; (2) no prior chromatographic separation and (3) no chemical derivatization. Furthermore, as an analytical technique NMR is robust and highly reproducible, it can be absolutely quantitative, it can be used in the precise structural determination of unknown metabolites, and it can be almost fully automated (Emwas 2015; Gonzalez-Gil et al. 2015; Li et al. 2016).

On the other hand, NMR spectroscopy itself and the analysis of complex biological mixtures by NMR is not trivial (Tiziani et al. 2008; Hajjar et al. 2017). In particular, the ^1^H NMR spectra of samples such as urine are very complex, typically consisting of > 1000 detectable and often overlapping peaks. The position, intensity and spectral width of these peaks is highly dependent on the number and types of chemicals in the mixture, the corresponding spin-coupling patterns of those chemicals and a wide variety of sample parameters. These parameters include: sample pH, sample salt type and salt concentrations, dissolved oxygen content, the presence of paramagnetic ions, the choice of solvent(s), temperature, temperature gradients, spectrometer field homogeneity, and primary magnetic field strength (to name just a few). In addition to the sample characteristics, NMR setup and processing parameters can also have a significant impact on the quality of NMR spectra and their subsequent interpretation. The choice of the pulse sequence for data acquisition, the selection of an appropriate solvent suppression technique, the level of decoupling power, the type of chemical shift reference(s), the length of the 90° pulse, the number of data points collected, the repetition time, receiver gain, the quality of shimming, the quality of tuning, and the number of acquisitions will all have a significant impact on the quality of NMR spectra and the presence of peak distortions or anomalies. Similarly, spectral processing choices concerning the extent of zero filling, choice of digital filters, selection of apodization functions, precision of the chemical shift referencing protocol, accuracy of the phasing, and the quality of baseline correction will also affect the results. Detailed suggestions and recommendations for handling many of these parameters, especially for NMR-based studies of urine, have been given in several recent reviews (Emwas 2015; Emwas et al. 2016).

Using these consensus recommendations, it should now be possible for almost anyone with a high-field NMR instrument to collect and generate (automatically or semi-automatically) high quality 1D ^1^H spectral data from complex biofluids. However, there is still relatively little consensus in the community regarding what to do after the NMR spectra are collected—i.e. the post-processing steps. Two “camps” have emerged in the field of NMR-based metabolomics. One camp tends to use spectral deconvolution software to identify and quantify compounds in individual NMR spectra. In this approach, each NMR spectrum is analysed individually and the resulting compound IDs and concentrations from multiple spectra are compiled to create a data matrix for multivariate statistical analysis. A variety of software tools for NMR spectral deconvolution have been developed including the Chenomx NMR Suite (Mercier et al. 2011), Bruker’s AMIX (Czaplicki and Ponthus 1998), Bruker’s JuiceScreener (Monakhova et al. 2014) and WineScreener (Spraul et al. 2015), Batman (Hao et al. 2014), and Bayesil (Ravanbakhsh et al. 2015).

The second camp uses statistical approaches to initially align multiple NMR spectra, to scale or normalize the aligned spectra, and then to identify interesting spectral regions (e.g. binning) or peaks that differentiate cases from controls (Smith et al. 2009; Barton et al. 2008; Lindon et al. 2007; Beckonert et al. 2007a). This approach, which is often called statistical spectroscopy, performs compound identification or quantification only after the most interesting peaks have been identified. This final identification step may use spectral deconvolution, compound spike-in methods or peak look-up tables (Martinez-Arranz et al. 2015). A variety of software packages for NMR statistical spectroscopy have been developed including, MetAssimulo (Muncey et al. 2010), Automics (Wang et al. 2009), Statistical total correlation spectroscopy (Cloarec et al. 2005a, b), and MVAPACK (Worley and Powers 2014).

For relatively simple biofluids such as serum, plasma, cerebrospinal fluid (CSF), fecal water, juice, wine or beer, NMR spectral deconvolution approaches appear to work very well (Ravanbakhsh et al. 2015). Extensive spectral libraries now exist for many of these biofluids and a number of the deconvolution software tools are becoming almost fully automated. Indeed, some software packages can be extremely fast and robust with compound coverage easily exceeding 90% and compound quantification errors often below 10% (Worley and Powers 2014; Zheng et al. 2011; Hao et al. 2014; Mercier et al. 2011; Ravanbakhsh et al. 2015). On the other hand, for very complex biofluids such as cell growth media, cell lysates and urine, the corresponding NMR spectra are often too complex for spectral deconvolution (manual or automated). The compound coverage rarely exceeds 50% and the level/quality is highly dependent on the skill and/or experience of the operator. There are also several reports showing considerable discrepancies between different laboratories (Sokolenko et al. 2013) or different users when spectral deconvolution is applied to very complex biofluids. As a general rule, for the routine analysis of urine 1D ^1^H NMR spectra, statistical spectroscopy techniques presently appear to be the best option. These approaches are robust and they allow useful results to be obtained with relatively little manual effort. They also facilitate the identification and quantification of key compounds or features in NMR-based urine metabolomic studies.

**Fig. 1:**
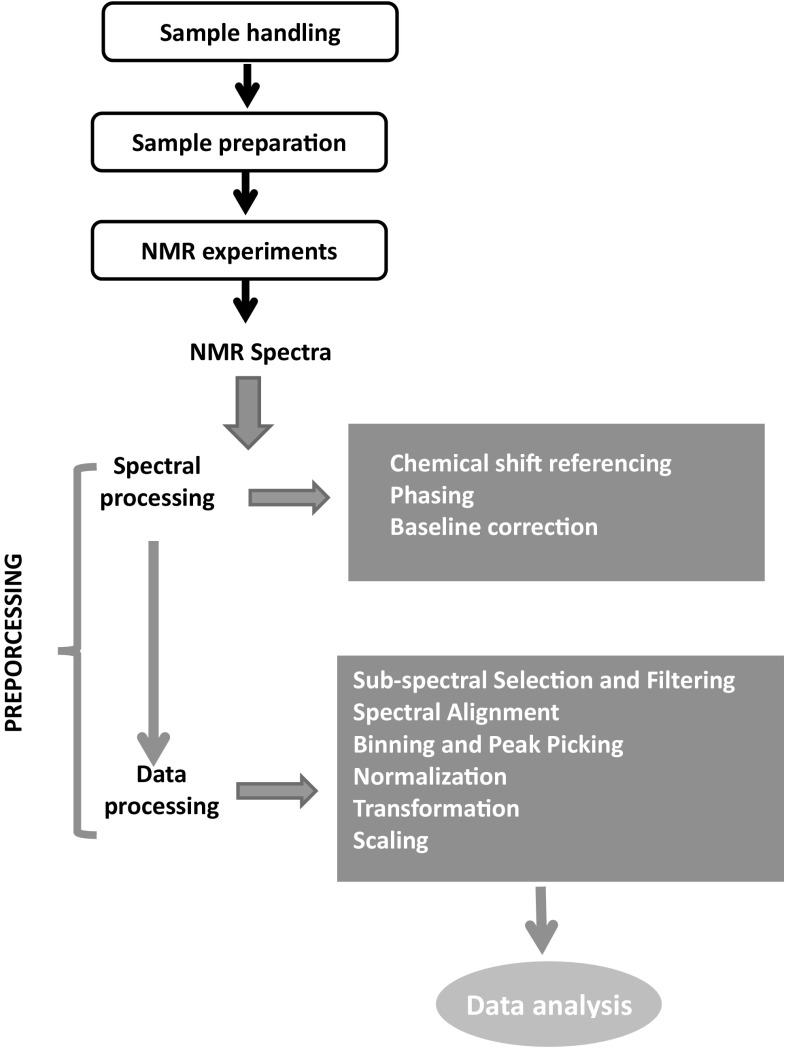
Summary of spectral processing and post-processing steps on urinary NMR-data

The purpose of this review is to assess and provide consensus recommendations for the processing of NMR data of biofluids with a particular focus on urine. NMR data processing refers to both spectral processing and data processing, as summarized in Fig. 1. In particular, we will review and discuss consensus recommendations for spectral processing, namely chemical shift referencing, phasing and baseline correction. These steps are critical for generating high quality NMR data. The remainder of this review will focus on providing recommendations for “post processing” of NMR data, including the determination of interesting spectral regions (alignment and binning) as well as spectral normalization, scaling and transformation. These are critical steps to statistical spectroscopy and their correct implementation is essential to the successful NMR analysis of urine (and other biofluid) samples.

## Spectral processing

### Chemical shift referencing

**Fig. 2:**
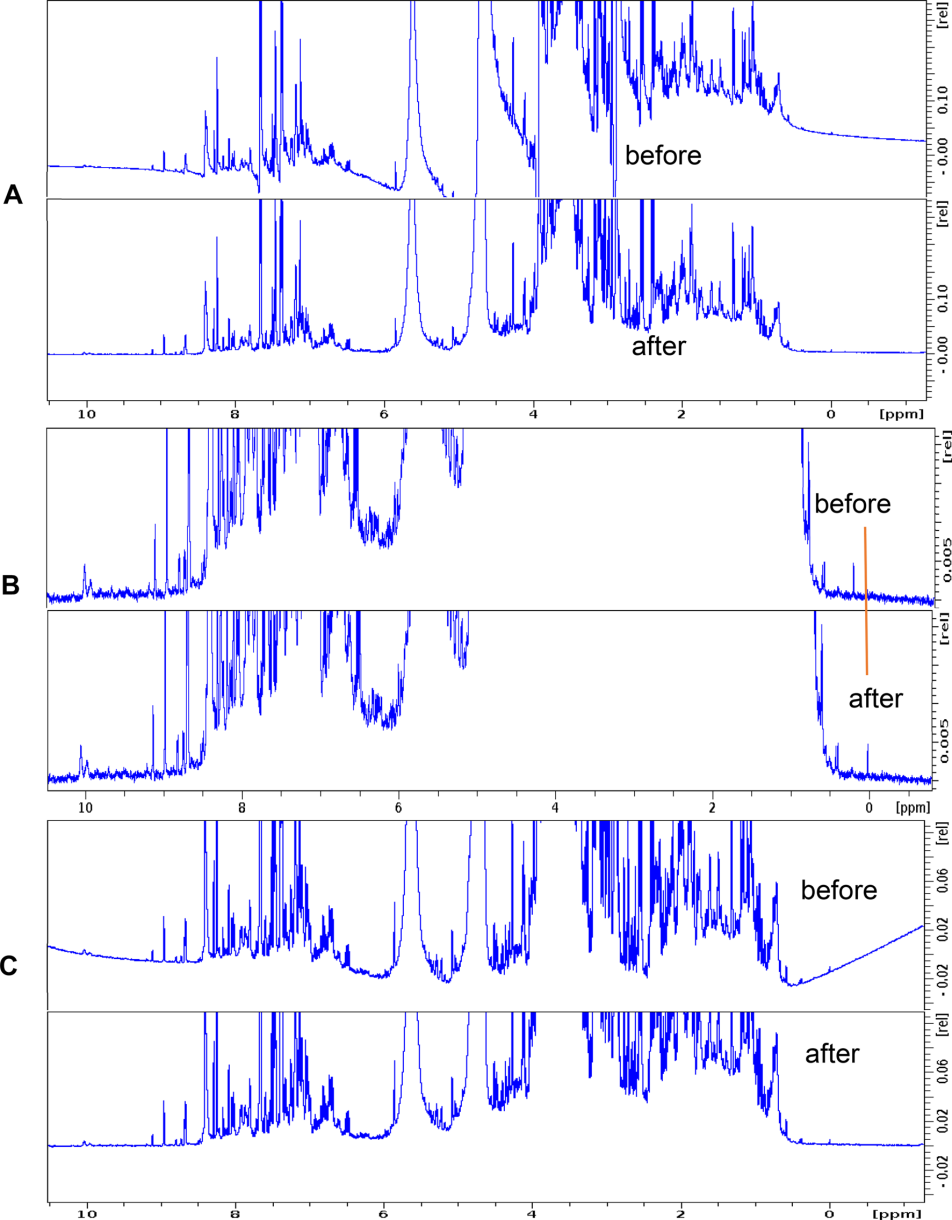
A simple visualization of the effects of **a** phasing, **b** referencing and **c** baseline correction and on an NMR spectrum

As any good NMR spectroscopist knows, NMR spectra must always be properly referenced using an internal chemical shift standard (Emwas 2015; Emwas et al. 2016; Harris et al. 2008a, b; Nowick et al. 2003). Chemical shift referencing is important for compound identification, for peak alignment and any multivariate statistical analyses that may follow Fig. 2. Within the metabolomics community both 4,4-dimethyl-4-silapentane-1-sulfonic acid (DSS) and 3-(trimethylsilyl)-2,2′,3,3′-tetradeuteropropionic acid (TSP) are widely used as chemical shift reference standards (Donaa et al. 2016). However, it is important to note that TSP is actually quite pH sensitive (Wishart et al. 1995).

This pH sensitivity can wreak havoc with spectral alignment, especially if samples have not been well buffered and/or carefully pH corrected. Therefore, we strongly recommend the use of DSS (especially deuterated DSS) as the chemical shift reference standard for biofluid (esp. urinary) NMR spectroscopy. We note that DSS is the chemical shift standard recommended by the IUPAC, IUPAB and IUBMB for biomolecular NMR (Markley et al. 1998). Chemical shift standards, such as DSS, can also be used for quantification, especially if the reference compound concentration is known precisely (Mercier et al. 2011). However, in biofluids such as plasma or serum, where DSS or TSP may become bound to macromolecules (proteins or lipoproteins), random variations in the reference intensity may occur, leading to inaccurate concentration estimates (Pearce et al. 2008). In these cases, an alternative internal standard for quantification (such as sodium acetate or sodium formate) is recommended. The use of the solvent water peak (i.e. H_2_O, and HDO in rapid exchange with non-observed D_2_O) for chemical shift referencing is very strongly discouraged since the signal position is sensitive to a wide variety of sample parameters, including temperature, pH, exchangeable moieties, salts and demagnetization field effects (Edzes 1990; Levitt 1996).

### Phasing

Phasing is a NMR spectral adjustment process that is intended to maximize the absorptive character and the symmetry of all NMR peaks over all regions of an NMR spectrum. Phasing is one of the most important steps in spectral processing as even small phasing errors can lead to significant problems that will ripple down through all remaining spectral processing and post-processing steps Fig. 2. In particular, phasing errors can affect spectral alignment, spectral binning and the measured peak areas (Wishart 2008). Even though automatic phasing is available in most modern NMR spectrometers, manual phasing is often required in metabolomics studies since many auto-phasing routines will distort low-intensity peaks. Phasing is particularly important for handling the residual (but often still prominent) water signal. A phase distortion in the solvent signal can substantially perturb the surrounding regions (~ 4.7 ppm). Auto-phasing programs may sometimes distort the entire NMR spectrum while attempting to correct for the residual solvent signal. Exclusion of the solvent region from auto-phasing procedures may help reduce this problem, however, manual phasing generally gives better results. Despite these caveats, auto-phasing is still widely used in the metabolomics community. This is because it is fast (allowing greater throughput) and it avoids operator bias.

We recommend that auto-phasing should be used as an initial phasing step. Subsequently, all NMR spectra should be manually inspected for phase distortions and, if necessary, those spectra exhibiting phase distortions should be phased manually. During manual phasing, the vertical scale should be increased as much as possible to allow for proper adjustment of the smaller signals. Even when manual phasing is performed by an experienced operator there are still some cases where it fails to improve spectral quality. Errors in executing or optimizing pulse sequence parameters can be manifested in some “phase-recalcitrant” spectra. The only way to correct for these problems is to re-acquire the spectrum using a standardized pulse sequence and using correct instrument parameters. Careful testing of a new pulse sequence’s performance on known, standardized samples (e.g. DSS with 90% H_2_O/10% D_2_O with several known small molecules in various spectral regions) is often necessary to ensure that any undetected or phase-distorting pulse-sequence errors will not propagate into the NMR spectra collected for “real” biofluids. In many cases, timing errors in the pulse sequence and/or instrument delays not properly taken into account are the main culprits leading to phase-recalcitrant spectra. These can be difficult to track down, but it is essential that they be detected and dealt with prior to acquiring a large number of spectra.

### Baseline correction

Baseline correction is another spectral processing technique that is critical for removing spectral artefacts that can arise from electronic distortions, inadequate digital filtering or incomplete digital sampling. When properly done, baseline correction yields a more pleasant looking spectrum where signal-free regions are completely flat, horizontal lines with zero intensity Fig. 2. While baseline correction is trivial for simple spectra with just a few peaks, it is somewhat more difficult for NMR spectra containing thousands of peaks with large differences in intensities (as is seen in urine). Correct baselines are critical for proper spectral alignment and proper peak integration (i.e. relative and absolute quantification). Small errors in the baseline structure can easily lead to errors (by orders of magnitude) in the quantification of low abundance metabolites. We recommend that all NMR spectra should be manually inspected for baseline distortions and, if necessary, those spectra exhibiting baseline distortions should be corrected using high quality baseline correction software.

Baseline correction in NMR is normally done via semi-automatic approaches that involve manual identification of reliable baseline regions followed by a computer-generated spline fit. Just as with phasing, baseline correction requires that the vertical scale should be increased as much as possible to allow for proper detection of those baseline regions needing correction. Software from all the major NMR vendors along with many third party software packages, such as NMRPipe (Delaglio et al. 1995), Chenomx NMR Suite (Mercier et al. 2011), or MestreLab Inc.’s MNova (to name just a few), can perform high quality baseline correction. All of these packages work in a semi-automated fashion, meaning that the baseline regions are first identified manually and then the programs complete the remaining baseline correction process. This correction process may use either time domain methods or frequency domain methods (Xi and Rocke 2008; Marion and Bax 1988; Halamek et al. 1994; Bao et al. 2012; Golotvin and Williams 2000; Wang et al. 2013; Bartels et al. 1995). We recommend the frequency domain correction methods as they are more widely used. Frequency domain methods attempt to construct a new baseline curve within the processed spectra directly using techniques such as asymmetric least squares (Peng et al. 2010; Eilers 2003), regular polynomial fitting or spline curve fitting and iterative polynomial fitting with automatic thresholding (Feng et al. 2006). More recently, a parametric approach that employs weighted scatter plot smoothing (LOWESS) has been used to estimate noise levels and generate more accurate baselines for metabolomic studies (Xi and Rocke 2008).

Fully automated baseline correction has been implemented in certain packages such as Bayesil (Ravanbakhsh et al. 2015) and MestreLab’s MNova suite, but these methods are currently limited to simpler biofluid spectra of serum, plasma, fecal water or cerebrospinal fluid. If and when fully automated methods appear for urine analysis, we would recommend them over manual methods as these automated methods would remove any user bias in baseline correction.

## Data post-processing

Data post-processing refers to the steps involved in assessing processed NMR spectra prior to the identification and comparison of important peaks and peak intensities. As mentioned in the introduction, NMR spectra of urine (or other very complex biofluids with > 75 detectable metabolites) require some degree of spectral simplification. This simplification can be achieved through several data post-processing steps: (1) sub-spectral selection; (2) spectral alignment; (3) spectral binning to extract peak intensities; (4) scaling and normalization, and finally (5) important peak identification (via multivariate statistics). Together, these approaches allow users to identify and quantify the most informative peaks in a given biofluid or urine NMR spectrum.

### Sub-spectral selection and filtering

Sub-spectral selection is a filtering technique involving the selection of only the interesting regions and discarding the uninformative areas of a given NMR spectrum. In general, not all parts of a recorded NMR spectrum are important for identifying and quantifying metabolites. For instance, in urine, the region between 0.00 and 0.60 ppm can be safely removed before alignment and/or binning since no metabolite signals (except possibly those from vacuum grease and other contaminants) exist in this portion of the spectrum. The water signal region from 4.50 to 4.90 ppm is also commonly excluded, as the residual solvent signal after suppression is not of interest and often interferes with the analysis of other metabolites signals. In urine samples, urea is one of the most highly concentrated metabolites and its peak is relatively close to the water resonance (near 6.00 ppm). Urea’s exchangeable protons are significantly affected by most water suppression techniques and so urea’s signal intensity changes significantly with the degree or quality of water suppression. Therefore, the urea peak (and the surrounding region, if affected) is normally excluded from further analysis. To summarize, we recommend the removal of the upfield region (0.00–0.60 ppm), the residual water region (~ 4.50 to 4.9 ppm) and the urea region (5.5–6.1 ppm) when analysing urine NMR spectra.

### Spectral alignment

Spectral alignment is a process that iteratively shifts peak positions in multiple spectra so that the peaks corresponding to the same compounds can be directly overlaid or aligned. Spectral alignment is needed to ensure that the same peaks, from the same compounds, can be compared and quantified across multiple NMR spectra. Signals or peaks that are inconsistently shifted across different NMR spectra, will not be properly matched and subsequent binning steps, scaling steps and multivariate analysis of the binned/scaled intensities will be compromised. While spectral alignment is widely used in NMR spectral analysis, it is also important to remember that alignment can hide important information encoded in chemical shift data, including sample pH, metal ion concentrations, ionic strengths and temperature.

**Fig. 3:**
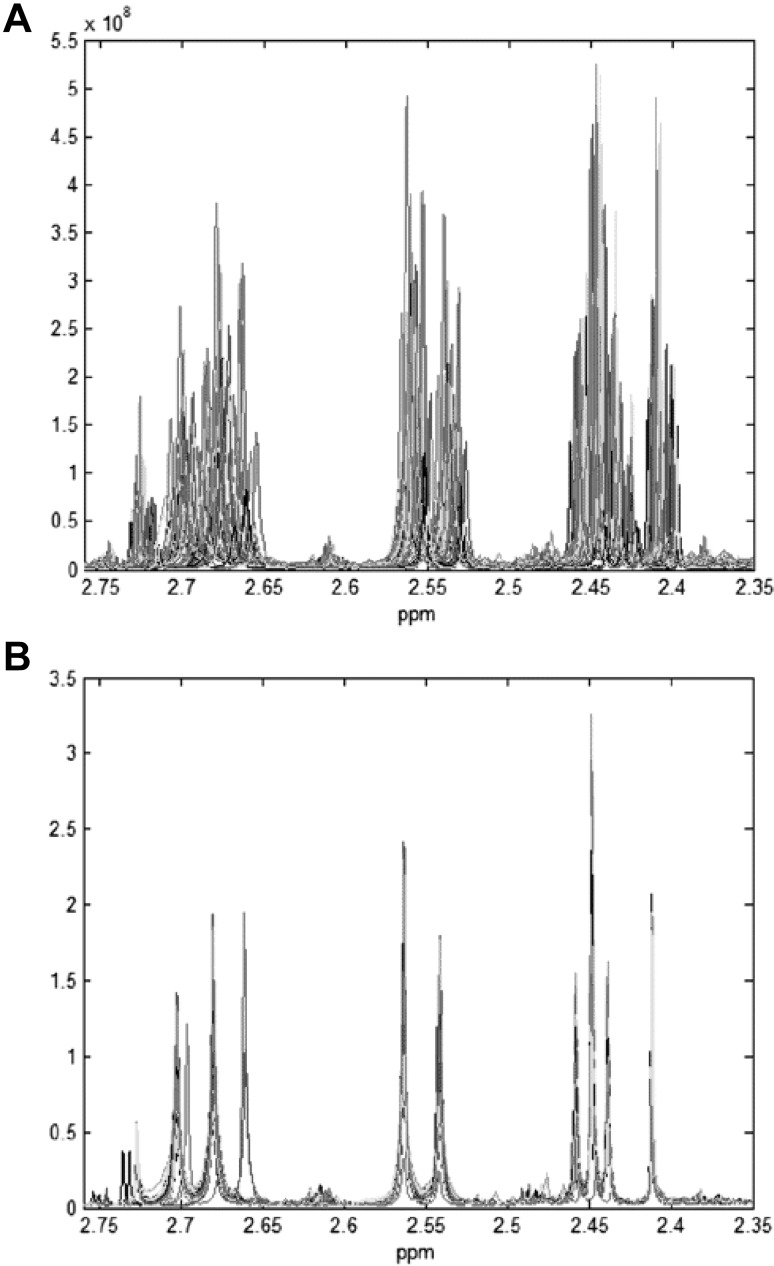
NMR spectra of urine samples, **a** original spectra in the selected region, and **b** normalized spectra warped to spectrum number 52 in the same region, from (Wu et al. 2006a)

Spectral alignment is trivial for NMR spectra with a small number (< 20) of peaks. However, it is not trivial for NMR spectra with thousands of peaks as is frequently seen for NMR spectra of biofluids such as urine. Even when properly referenced, the chemical shifts of many compounds in urine are often subject to a phenomenon known as chemical shift drift (Giskeodegard et al. 2010; Wu et al. 2006a), which is shown in Fig. 3. Chemical shift drift is an environmental effect that can be due to several factors such as sample pH, ionic strength, changes of temperature, instrumental factors, level of compound dilution and relative concentration of specific ions (Defernez and Colquhoun 2003; Cloarec et al. 2005b). The net result of chemical shift drift is that it is often quite difficult to determine which peaks match to which compounds when comparing one urine spectrum to another. One experimental approach to address chemical shift drift is to precisely control the pH and salt concentration of the sample by adding a strong buffer solution to the sample (pH 7.0, 400 mM phosphate, 20–30% by volume). However, this is often not practical for large numbers of samples and it may not always correct other ionic contributions to chemical shift drift. As a result, several computational methods have been developed to correct the movement of NMR peaks. These are called peak alignment or spectral alignment methods and they include such processes as correlation optimized warping (COW) (Nielsen et al. 1998), fuzzy warping (Wu et al. 2006b), peak alignment by beam search (Forshed et al. 2003; Lee and Woodruff 2004), and interval correlation shifting (icoshift) (Savorani et al. 2010). These methods are known as pairwise alignment techniques because they align each NMR spectrum to a chosen reference NMR spectrum, one by one. The reference spectrum can either be real or virtual and should always be representative for the whole dataset. More details about these spectral alignment algorithms are given below.

COW is an older alignment approach developed in the late 1990s that uses a technique called segment warping (Tomasi et al. 2004). More specifically, COW is a piecewise or segmented data preprocessing method (where the spectrum is divided into equal sized segments) aimed at aligning a sample spectrum towards a reference spectrum by allowing limited changes in segments lengths on the sample spectrum. This method was originally designed to be used for the alignment of chromatographic data, but it has proven to be useful for the alignment of NMR spectra as well (Tomasi et al. 2004; Smolinska et al. 2012b).

The Beam search method for peak alignment of NMR signals was developed in the early 2000’s based on genetic algorithms for optimization (Lee and Woodruff 2004; Forshed et al. 2003). In this method each spectrum is divided into a number of segments then each segment is aligned to a corresponding region in a reference spectrum using a genetic algorithm (Forshed et al. 2002). A smaller part of the spectrum (covering a region spanning ~ 0.15 ppm) is aligned to a corresponding reference by shifting (right or left) and then using linear interpolation to adjust the spectra piecewise (Forshed et al. 2003).

Another technique for NMR peak alignment is called the fuzzy warping method which was originally developed and used for the alignment of urine NMR spectra (Wu et al. 2006a). Fuzzy warping seeks to establish a correspondence between the most intense peaks in the spectra to be aligned, where iterative procedures alternate between fuzzy matching and signal transformation. The parameters are weighted according to the corresponding of target spectrum. The performance of the peak alignment can be carried out to assess the alignment procedure in terms of any erroneous alignment or change of peak shape (Wu et al. 2006a).

The interval correlated optimized shifting (icoshift) method is the newest approach to NMR spectral alignment (Savorani et al. 2010). It is based on dividing a given NMR spectrum into different segments or intervals, then aligning the spectral intervals to the corresponding segment of a reference spectrum. Icoshift optimizes the piece-wise cross-correlation using a fast Fourier transform (FFT) and a greedy algorithm that allows for user-defined recursion. In particular, each spectrum or interval is allowed to shift right or left until the maximum correlation to the target spectrum is achieved. The use of the FFT approach allows for simultaneous processing and alignment of all spectra. Icoshift has been found to be substantially faster than other algorithms (such as COW, fuzzy warping and beam search) thereby making full-resolution alignment of large 1D ^1^H-NMR datasets possible in just a few seconds—even on a desk-top computer. Unlike most other tools, icoshift also allows users to customize peak shape, peak multiplicity, peak position and peak height to better match the target spectrum. Icoshift is available as both an open source MatLab package and a Python package. While icoshift only achieves local alignment optimization and it cannot deal with strongly overlapped regions, the fact that it is open access and substantially faster/better than previously published methods, we recommend that icoshift should be used in the alignment of biofluid (esp. urine) NMR spectra.

Table 1 summarizes the four spectral alignment algorithms discussed above. A much more detailed discussion and assessment of NMR spectral alignment algorithms is provided in a recent review (Vu and Laukens 2013). While icoshift goes a long way towards simplifying and improving the quality of NMR spectral alignment, a fully automated, perfectly functioning NMR spectral alignment tool is still not available. In particular, the problem of peak order changes (Csenki et al. 2007) has yet to be addressed, as all existing alignment methods assume the same peak order between spectra.

**Table 1:** List of the alignment methods and presents their features

Short name	Full name	Technique	Target function	Peak picking based	NP	SW	Pair-Wise(#)	Correction method	Software	Remarks
COW	Correlation Optimized Warping (Nielsen et al. 1998)	Dynamic programming	Pearson correlation coefficient	No	2	Yes	Yes	Insert and deletion	(1)	Slow
PABS	Peak alignment by Beam search (Lee and Woodruff 2004)	Beam search algorithm	Pearson correlation coefficient	No	3	Yes	Yes	Shift & Insert and deletion	(+)	
FW	Fuzzy Warping (Wu et al. 2006a)	Fuzzy logic for matching most intense peaks	Maximize fuzzy membership Gaussian function	Yes	1	No	Yes	Insert and deletion	(4)	
icoshift	Interval correlation shifting (Savorani et al. 2010)	Segmentation model by equal size segments or manually selecting segments	FFT cross-correlation	No	2	Yes	Yes	Shift & Insert and deletion	(6)	The peak-piking step adds complexity

### Binning and peak picking

The next “post-processing” step is usually some form of binning. Binning can be a very simple method, not even requiring alignment, to extract peak intensities from multiple NMR spectra prior to performing multivariate statistical analysis. Binning involves dividing NMR spectra into small regions (typically spanning 0.04–0.05 ppm), which are sufficiently wide to include one or more NMR peaks. The intensity of each bin is determined by calculating the area under the curve (AUC). As a result, a typical urine NMR spectrum will often generate 500–1000 bins with non-zero intensities. Multivariate statistical analysis is then carried out on the extracted bin intensities and the most significant peaks (or bins) are then assigned to specific metabolites. Binning can be done using prior knowledge (i.e. knowing where metabolite peaks appear) or naively using an automatic algorithm.

Table 2 describes a number of common binning techniques including equidistant (equal size) binning (Izquierdo-Garcia et al. 2011), Gaussian binning (Anderson et al. 2008), adaptive-intelligent binning (De Meyer et al. 2008), dynamic adaptive binning (Anderson et al. 2011), adaptive binning using wavelet transforms (Davis et al. 2007) and an optimized bucketing algorithm (Sousa et al. 2013). Equidistant binning takes a spectrum and then divides it into equal spectral widths (i.e. 0.02, 0.04 or 0.05 ppm) and is the most commonly used binning method (Craig et al. 2006; De Meyer et al. 2010; Izquierdo-Garcia et al. 2011). However, a disadvantage of this method is the lack of flexibility with regard to boundaries in cases where peaks are split between two adjacent bins. Other methods such as adaptive-intelligent binning (De Meyer et al. 2008), dynamic adaptive binning (Anderson et al. 2011) and adaptive binning using wavelet transforms (Davis et al. 2007) can be utilized to overcome this problem by adjusting the bin position so that one bin can only cover complete peaks. We cannot recommend a single binning method because all of them have pros and cons, and their efficiency is somewhat dataset-dependent. As a general rule, equidistant binning is the most commonly used method (Smolinska et al. 2012a), and often works quite well despite its simplicity.

**Table 2:** Summary of binning methods with a brief description of the main features of each method

Method	Description	Remarks	References
Equal size binning	Spectrum is divided in simple rectangular regions of the same size. Each bin span the same number of spectral points	Straightforward and fast to apply. Works quite well despite simplicity. The bins size in ppm needs to be specified (0.04, 0.02, 0.01 ppm the most common choices). Peaks can be splitted across multiple bins	Izquierdo-Garcia et al. (2011)
Gaussian binning	A Gaussian kernel weights the signal contribution relative to distance from bin center, and the overlap between bins is controlled by the kernel standard deviation	Overlapping bins are used. Very robust to peak shifts. Two parameters (not easy to tune): standard deviation and step size that make a trade-off between loose of information and robustness	Anderson et al. (2008)
Adaptive-intelligent binning	Iterative algorithm that uses variable bin sizes adaptively inferred from spectra	No arbitrary parameters, reference spectra, a priori knowledge, or data modifications are required. Low-intensity peaks could be troublesome. Noise regions need to be specified	De Meyer et al. (2008)
Dynamic adaptive binning	Bin boundaries are determined by optimizing an objective function using a dynamic programming strategy. The objective function measures the quality of a bin configuration based on the number of peaks per bin	Ability to create bins containing a single peak. Two main parameters and several other parameters for peaks identifications	Anderson et al. (2011)
Adaptive binning using wavelet transform	Wavelet transforms are used to detect peaks in a reference spectrum. Integration is then performed over these peaks in each of the sample spectra. What constitutes a peak is determined by the amount of smoothing implicit in the wavelet transform	Noise regions are excluded. The amount of smoothing depends on the number of levels of the wavelet transform and can be adjusted according to the data resolution and the shifts expected between samples	Davis et al. (2007)
Optimized bucketing algorithm	A bucketing method that optimizes bucket sizes by setting their boundaries at the local minima determined through the average NMR spectrum	A mathematically simple approach. Two parameters need to be chosen, requiring visual inspection of the result	Sousa et al. (2013)

Several non-binning methods such as spectral deconvolution (Weljie et al. 2006), curve-fitting (Bollard et al. 2005), direct peak fitting (Schuyler et al. 2015), and peak alignment have been developed to overcome the drawbacks to binning. However, these methods are generally best for simpler biofluids (serum, plasma, CSF, saliva) and are not yet suited to handling the spectral complexity of urine.

### Normalization

After NMR peaks have been aligned, identified or binned, and their respective intensities determined, the next step in the post-processing pipeline is to correct for inherent concentration differences. Plasma and serum are examples of biofluids that are under strict physiological control, so the spectra collected from these biofluids (at least for the same organism) can often be compared without further adjustment, normalization or scaling. On the other hand, most other biofluids are not under such strict physiological controls and so corrections for dilution effects must be made, for example urine is certainly subject to substantial metabolite concentration variation. Urine volume varies greatly with fluid intake and it is also affected by many other physiological and pathophysiological factors. More specifically, the concentrations of endogenous metabolites in urine (even from the same individual) can vary by several orders of magnitude (Emwas 2015). Therefore, proper adjustment to accommodate these large intensity/concentration variations is critical. The best approach for doing this is called normalization, a well-known data processing technique that aims to make all samples comparable to each other. Note that normalization can mean different things under different situations. In statistics, normalization means transforming a collection of data so that it is normally distributed (i.e. follows a Gaussian distribution). In clinical science, normalization means multiplying the data by some correction factor to make the values more comparable. In this regard, normalization for clinical scientists is similar to the statistical definition of scaling.

**Fig. 4:**
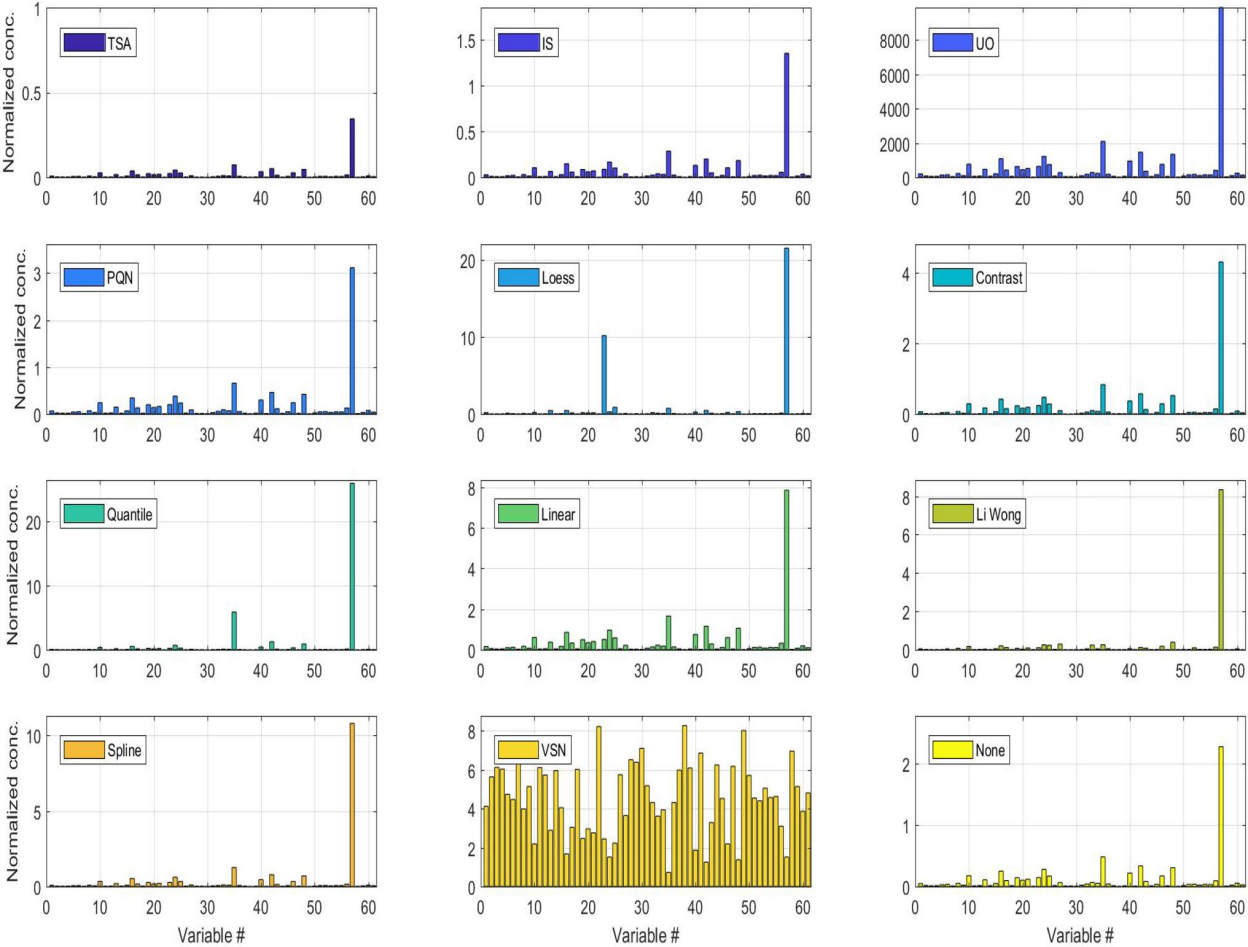
Metabolite concentrations in a urine sample after different normalization procedures have been applied. For a full description of the methods see Table 3. The data are originally from (Lusczek et al. 2013b) and were retrieved at http://www.ebi.ac.uk/metabolights/MTBLS123

Many approaches for sample normalization of urine have been proposed and reviewed in the literature (see Table 3). As a general rule, sample-to-sample normalization can be divided in two broad categories: physiological (normalization to the urine output relative to creatinine or osmolality) or numerical (i.e. all the others). Fig. 4 shows how metabolite concentration profiles change when different normalization strategies are applied to the data. Physiological normalization generally requires a separate measurement using: (1) an osmometer (or osmality meter) to measure the electrolyte to water balance, (2) a refractometer to measure refractive index (a proxy for specific gravity) or (3) a creatinine test (via direct measurement using an enzyme assay or by NMR analysis/integration of the creatinine peaks). Physiological normalization (especially to creatinine) is how most urine concentrations are reported in the clinical and biochemical literature. Its widespread use in the medical community made it a preferred normalization option in the past. However, normalization to creatinine assumes that creatinine clearance is constant and this may not be true in presence of metabolic dysregulation. Therefore, normalization to creatinine should be used only when significant metabolic dysregulation is not suspected (which is not always the case). Measures of urinary specific gravity and osmolality are not as highly dependent on the state of an individual’s metabolic regulation. As a result they are gaining increasing traction in the urinalysis community(Miller et al. 2004; Edmands et al. 2014; Sauve et al. 2015; Waikar et al. 2010; Tang et al. 2015). Therefore, for physiological normalization of NMR-based urinary metabolomic data we recommend the use of specific gravity over creatinine. However, physiological normalization assumes one is working with real concentration data (uM or mM) and in many cases with NMR-based urine metabolomics, only relative concentration data (i.e. no concentration units) are available.

**Table 3:** Characteristics of common normalization procedures. Descriptions are based on references (Hochrein et al. 2015; Kohl et al. 2012; Saccenti 2017)

Method	Abbr.	Description	Remarks	References
Urine output (Elizabeth R. Lusczek et al., 2013b)	UO	Urine output is the volume of urine excreted per hour per kilogram of mass. Normalization is performed by multiplying raw metabolite concentrations by urine output	Total urine output may be difficult to obtain	Lusczek et al. (2013a)
Osmolality	OSM	Osmolality is the concentration of solute particles in the urine. Normalization is carried on by dividing concentration by the osmolality of each sample, returning	It reflects physiological metabolite-concentrating mechanisms in the kidneys Require intensive lab work	Lusczek et al. (2013a)
Normalization to an internal standard	IS	The concentration of each metabolite is divided by the concentration of an internal standard, usually creatinine	Constant excretion of creatinine into urine (creatinine clearance) is assumed to be constant. Exact creatinine concentration may be difficult to estimate from NMR spectra	Jatlow et al. (2003)
Constant sum (1)	SC	Metabolites concentration are divided by the sum of the concentration for all the measured metabolites	Assumes that the concentration of each metabolite is increased by the same amount due to the increased concentration of the urine sample	Lusczek et al. (2013a)
Constant sum (2)	SC-LUG	Same as CS but lactate, glucose, and urea concentrations are excluded	Implies that lactate, glucose, and urea concentrations are highly variable during the experiment	Lusczek et al. (2013a)
Total spectrum area	TSA	The (binned) spectra are summed over the entire spectral area excluding regions containing water and urea resonances	See CS and CS-LUG	Lusczek et al. (2013a)
Probabilistic quotient normalization	PQN	It starts with an integral normalization of each spectrum, followed by the calculation of a reference spectrum (median or baseline). For each variable of interest the quotient of a given test spectrum and the reference spectrum is calculated and the median of all quotients is estimated. All variables of the test spectrum are then divided by the median quotient	It assumes that biologically interesting concentration changes influence only parts of the NMR spectrum, while dilution effects will affect all metabolite signals. Choice of reference spectrum is arbitrary	Dieterle et al. (2006)
Cyclic loess normalization	Loess	The log-transformed ratio of any two spectra is compared to their average feature by feature; then, a normalization curve is fitted using non-linear local regression (loess) and subtracted from the original values	Assumes the presence of non-linear biases, such as intensity-depended biases, is assumed	Dudoit et al. (2002; Cleveland and Devlin, 1988)
Contrast normalization	Contrast	By means of an orthonormal transformation the matrix into a transformed onto a contrast space. Normalizing curves are fitted similarly to those in Cyclic Loess Normalization, using a robust distance measure based on the Euclidean norm. The contrasts are evened out by a smooth transformation and data are mapped back to the original input space	Cyclic loess normalization	Åstrand (2003)
Quantile normalization	Quantile	All spectra are brought to an identical distribution of intensities across features (bins or metabolites). quantile	After QN the vectors of feature intensities consist of the same set of values, however, these values are distributed differently among features	Bolstad et al. (2003)
Linear baseline normalization	Linear	A scaling factor is used to map linearly from each spectrum to the baseline. The scaling factor is computed for each spectrum as the ratio of the mean intensity of the baseline to the mean intensity of the spectrum	It assumes a constant linear relationship between each feature of a given spectrum and the baseline Baseline is arbitrary: it can be constructed by calculating the median of each feature over all spectra	Bolstad et al. (2003)
Non-linear baseline normalization	Li-Wong	A normalization curve is fitted to map a spectrum to the baseline spectrum (having the median overall intensity) on a scatter plot. The normalization curve is fitted only on non-differentially expressed features which are used for finding the normalizing piecewise linear running median line	It is assumed that features corresponding to unregulated metabolites have similar intensity ranks in two spectra. Possible non-linear relationships between the baseline and the individual spectra are also assumed	Li and Wong (2001)
Cubic-spline normalization	Spline	A baseline spectrum is built by computing the geometric mean of the intensities of each feature over all spectra A set of evenly distributed quantiles is taken from both the target spectrum and the sample spectrum and used to fit a smooth cubic spline. The process is iterated several times shifting the set of quantiles by a small offset each time. Finally a spline function generator uses the generated set of interpolated splines to fit the parameters of a natural cubic spline	The existence of non-linear relationships between baseline and individual spectra are assumed The geometric mean can be substituted by the arithmetic mean for reasons of robustness to negative values	Workman et al. (2002)
Shapiro–Wilk	SW	Features showing high variability in concentration are iteratively removed until mostly nonregulated features remain. These are be used as reference features for subsequent data normalization	It assumes that the variance of regulated features across all specimens is larger than that of nonregulated features	Hochrein et al. (2015)
Linear mixed	LM	It fits a mixed model to metabolite concentration with simultaneous estimation of the correlation matrix	Assume data as coming from a larger pool cohorts; same for the batches and samples	Jauhiainen et al. (2014)
EigenMS		Estimating treatment effects with an ANOVA model; singular value decomposition of the residuals matrix is then used to determine bias trends in the data. The number of bias trends is then estimated via a permutation test and the effects of the bias trends are eliminated	Not tested on NMR data	Karpievitch et al. (2014)
Variance stabilization normalization	VSN	VSN approaches are set of non-linear methods that used to keep the variance constant over the entire data range, leading to roughly equal variable variance. Found to work well with NMR data	Found to work well with NMR data	Huber (2002)

When physiological normalization is not possible, numerical normalization is a viable alternative and, in some cases, can yield even better normalization results than physiological normalization. There is now a large body of literature covering numerical normalization techniques for urine analysis (see Table 3 for a list of methods, abbreviations, short descriptions and references). Different approaches work better for different situations. Lusczek et al. (2013b) found constant sum (CS), constant sum excluding lactate, glucose, and urea concentrations CS-LGU and total spectral area TSA normalized data appear to correlate well with each other. They also do a good job of representing NMR spectral intensities. probabilistic quotient normalization (PQN) normalized data was found to be moderately correlated with UO and osmolality (OSM) data and not with CS, CS-LGU and total spectral intensity (TSI) normalized data.

Kohl et al. (2012) recently reviewed and compared many of the more advanced numerical normalization methods. In particular, they tested the impact of these normalization methods on data structures and sample classification using NMR data from healthy and autosomal polycystic kidney disease (ADPKD) patients. They found only four methods (Loess, Quantile, Linear and Spline normalization) that were able to perform better than methods without normalization for the detection of differentially expressed metabolites. For the accurate determination of metabolite concentration changes, the same four methods provided the most uniform results for all tested metabolites investigated.

In a sample classification context, Quantile and Spline normalization were found to be the best performing methods. Overall, they found that Quantile normalization outperformed all of the most common normalization methods, but achieved mediocre classification performance for small data sets. The opposite was found for Spline normalization. In contrast, Filzmoser and Walczak (2014) found PQN to outperform other methods and recommended it over other numerical normalization techniques. However, Saccenti (2017) found that PQN did not perform particularly well in discriminant/classification setting (see the results of partial least squares discriminant analysis shown in Table 4).

**Table 4:** Quality of PLS-DA model for the discrimination of two groups when different normalization approaches are applied on the data. The table is reproduced with permission from (Saccenti 2017)

	Method	NMC	*Q* ^*2*^	*DQ* ^*2*^	AUROC
1	TSA	64	0.02	0.02	0.60
2	IS	77	− 0.33	0.005	0.37
3	UO	57	− 0.04	0.007	0.61
4	PQN	62	− 0.05	− 0.03	0.64
5	Loess	1	0.89	0.92	1
6	Contrast	23	0.41	0.56	0.99
7	Quantile	7	0.63	0.81	0.99
8	Linear	86	− 0.69	− 0.02	0.28
9	Li Wong	64	− 0.25	− 0.03	0.56
10	Spline	7	0.36	0.7	1
11	VSN	0	0.95	0.97	1
12	None	33	0.21	0.25	0.77

*NMC* Number of misclassifications, *DQ*^*2*^ discriminant Q^2^, *AUROC* area under the receiver operating curve. The measures are discussed in (Saccenti 2017)

It is interesting to note that total content normalization, urinary output normalization, internal standard normalization, and probabilistic quotient normalization were originally developed for processing metabolomic data. All of the other methods were developed to normalize microarray data, which have inherently different properties in terms of variance and covariance patterns and error structure. Indeed, the performance of the latter normalization methods on metabolomics data can be quite inconsistent, as observed by a number of different authors (Hochrein et al. 2015; Saccenti 2017).

Many of the numerical methods used for normalization implicitly assume that the average sum of measured metabolite concentrations is constant across samples or group of samples. In other words, it is assumed that the total quantity of dissolved metabolites is invariable. Unfortunately, this is often an unrealistic assumption. In particular, Hochrein et al. (2015) showed that commonly used normalization and scaling methods fail to retrieve true metabolite concentrations in the presence of increasing amounts of glucose added to simulate unbalanced metabolic regulation. They also proposed an alternative method to compensate for these effects in the presence of marked unbalanced metabolic regulation.

**Fig. 5:**
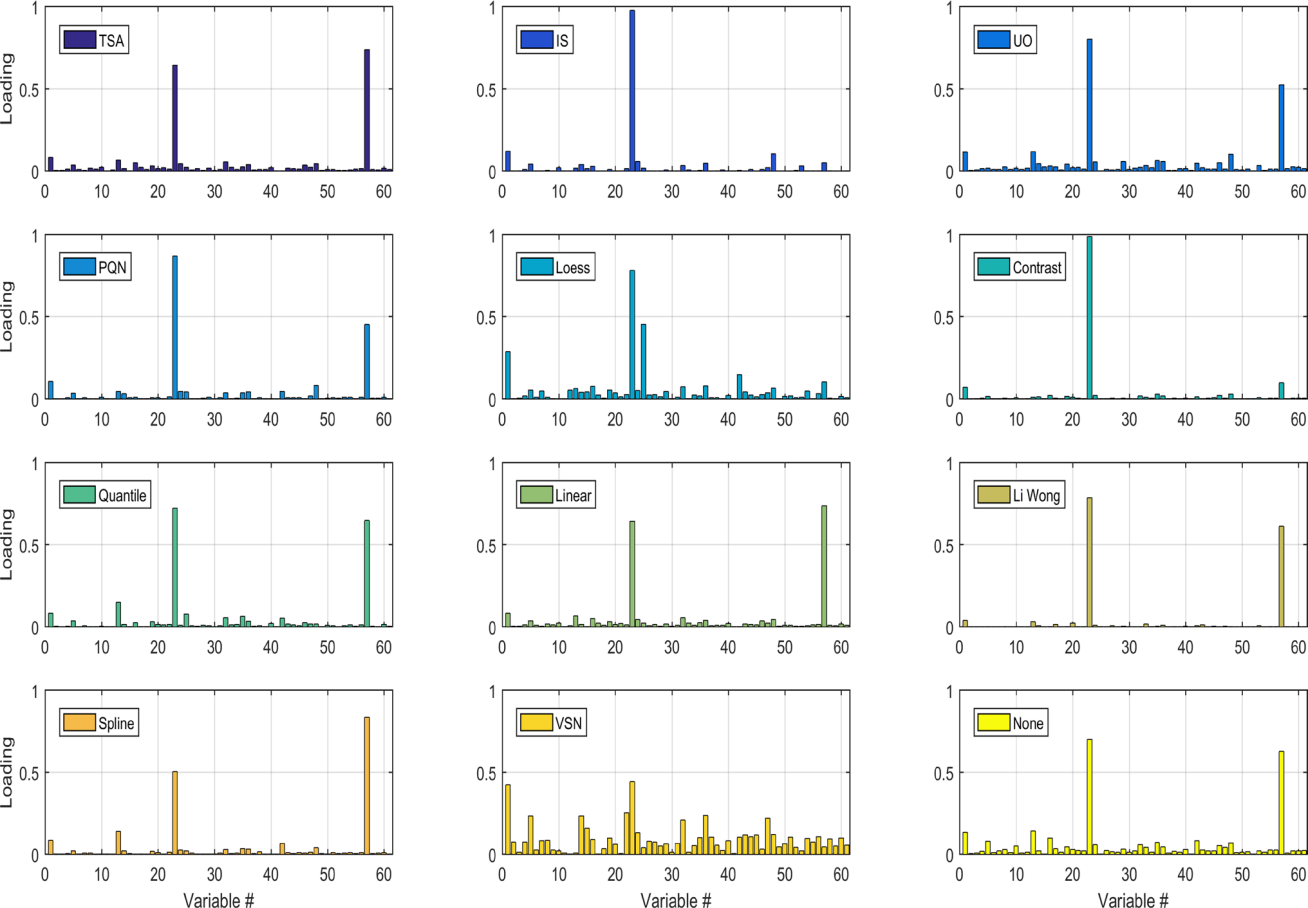
Loadings for the first principal components for a PCA model fitted on the data normalized with the different procedures. Data are Pareto scaled before PCA. ) Data are from (Lusczek et al. 2013b) and have been retrieved at http://www.ebi.ac.uk/metabolights/MTBLS123</link> The figure is from reference (Hochrein et al. 2015

All normalization methods alter the structure of the data and the results of subsequent analysis will be affected by the choice of the normalization method applied, especially when the data are used to infer correlations and biological networks as described in (Saccenti 2017). Jauhiainen et al. (2014) proposed a method based on linear mixed modelling, and found that it performed well when assessing robustness and its ability to discover true correlations. Figure 5 shows the results of a principal component analysis, which is one the most commonly used multivariate tools in metabolomics (Table 3), after it has been applied to the data. While this is just one example taken for one particular data set, it clearly illustrates how normalization not only affected the results of this exploratory analysis but also the performance of the methods used to discriminate between groups of samples, which is a typical problem in metabolomics studies.

It is evident from the reported literature that there is no consensus on which numerical method should be applied to normalize data and that a consensus is difficult to establish. Therefore, we are unable to make a formal recommendation on which numerical normalization method should be used for NMR-based urinary metabolomics. Based on the data at hand, it seems advisable to use PQN when the goal is biomarker selection but when the goal is discrimination/classification Quantile normalization for large (> 50 samples) data sets would seem to perform best, while Spline normalization seems to work better for smaller data sets.

### Scaling and transformation

Scaling and transformation refer to statistical techniques that help to make data more normally distributed or to reduce the spread in values by employing a mathematical operation on the spectral signal intensities (or concentrations) for all samples. As mentioned earlier, urinary metabolite concentrations can range over several orders of magnitude. The detectable variations in metabolites with higher concentrations will of course be easier to detect than the ones with low concentrations. This can lead to a bias or an undue influence from highly concentrated metabolites on the results of a urinary metabolomic study (Ebbels et al. 2011). This influence can, in turn, make a small number of metabolites dominate the outcomes from multivariate statistical analyses. To avoid this kind of bias it is often necessary to scale metabolite intensities before undertaking any further analysis (van den Berg et al. 2006). Table 5 shows a list of scaling and transformation methods, several of which were investigated and compared by (van den Berg et al. 2006). Centering is commonly used to adjust the differences between low-concentration and high-concentration metabolites by scaling all values so that they vary around zero (zero becomes the mean metabolite level). Mean-centering, on its own, is not sufficiently powerful to correct for scaling issues if the data is composed of sub-groups with different variability. As a result, mean centering is usually combined with other scaling methods.

**Table 5:** Summary of centering, scaling, and transformation methods based on reference (van den Berg et al. 2006), where $\overline {{{x_i}}}$ indicate the average and S_i_ the standard deviation

Method	Description	Drawbacks
Centering ${x_{ij}}={x_{ij}} - {\overline {x} _{ij}}$	In this method the mean is subtracted from each column. Mean of transformed variance will always be “zero”. It is usually applied in most data analysis methods like PCA and PLS regression	In heteroscedastic data, it is not always sufficient
Autoscaling ${x_{ij}}=\frac{{{x_{ij}} - {{\overline {x} }_{ij}}}}{{{s_i}}}$	In this method each column is centred by subtracting the mean from each peak intensity then divided by the standard deviation of that column. Makes all variable variances equal; important in PCA and PLS application to avoid that high variance variable dominate the model	All variables become equally important, even noise. This may be a problem in NMR applications where large portions of the spectra contain only nosie
Pareto scaling ${x_{ij}}=\frac{{{x_{ij}} - {{\overline {x} }_{ij}}}}{{\sqrt {{s_i}} }}$	Pareto scaling is similar to autoscaling but each column divided by square root of the standard deviation after mean centring. Good method to reduce the influence of intense peaks while emphasizing the weak peaks. Makes variable variance roughly equal. Well suited for the analysis of NMR data since it downgrade the importance of the nosie while preserving the variance structure of the data	Like autoscaling, sensitive to large fold changes but at less extent
Range scaling ${x_{ij}}=\frac{{{x_{ij}} - {{\overline {x} }_{ij}}}}{{\left( {{x_{i,\hbox{max} }} - {x_{i,\hbox{min} }}} \right)}}$	In this method each column centere to the mean, then divided minimum and maximum range of that particular metaboloite. Makes variable variance roughly equal; better for explorative analysis	Enhances variables with smaller variability; reduce consistently the variance. Very insensitive when the minimum and maximum range is very large
Level scaling ${x_{ij}}=\frac{{{x_{ij}} - {{\overline {x} }_{ij}}}}{{{x_i}}}$	In this method each column is centre to the mean then divide by the same mean. Makes variable variance roughly equal while preserving the variance structure. Suitable for biomarker identification	May be problematic for NMR data since it may inflate low value variables
Logarithmic transformation ${x_{ij}}=\log ({x_{ij}})$	Correct for heteroscedasticity and non-normality; pseudo scaling effects. Suited for concentration data and multivariate analysis	Enhances small values in the case of noisy NMR data. Not applicable to 0 values; ineffective in bi-modal data distribution
Power transformation ${x_{ij}}=\sqrt[n]{{{x_{ij}}}}$	Correct for heteroscedasticity and non-normality; pseudo scaling effects; applicable to 0 values. It is a good alternative to situation when logarithmic scaling is not possible	Choice of power is arbitrary; cannot be applied for negative values
Generalized log transformation ${x_{ij}}=\ln \left( {{x_{ij}}+\sqrt {x_{{ij}}^{2}+\lambda } } \right)$	Suitable for classification/discrimination applications	May suffer form numerical instability and not suitable in the case of very small values

**Fig. 6:**
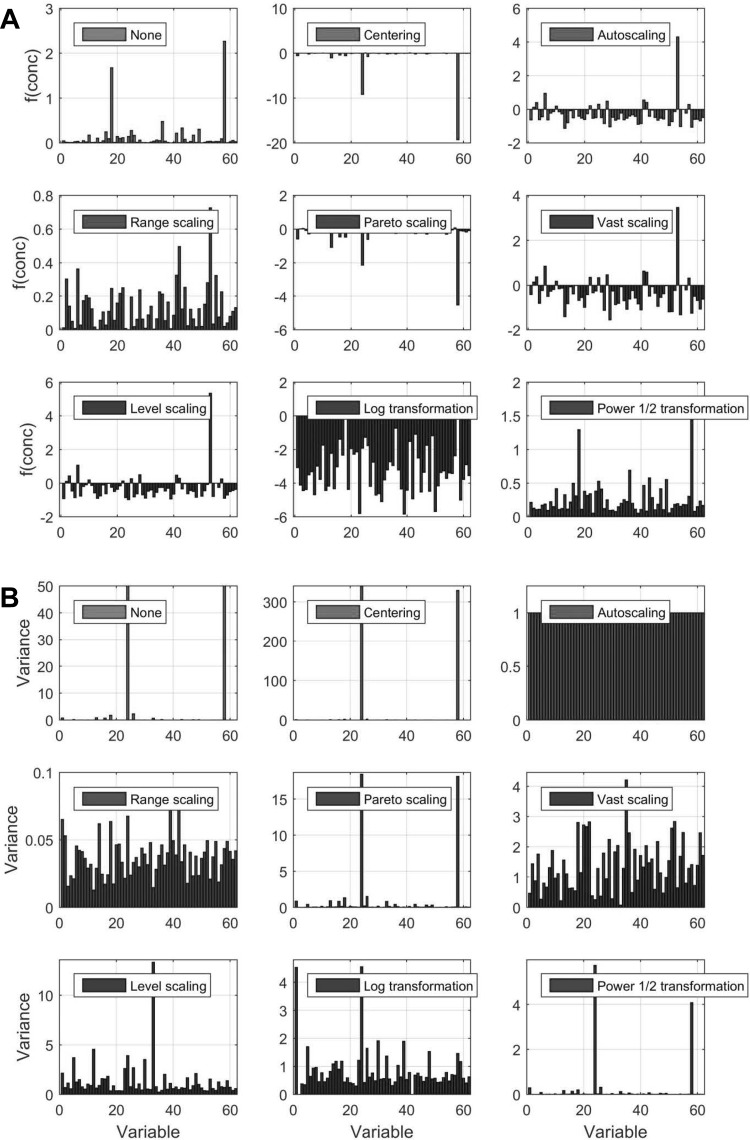
Effect of different centering, scaling and transformation approaches on concentration values (**a**) and variance (**b**). For a description of the methods see Table 4. Data are from (Lusczek et al. 2013b) and have been retrieved at http://www.ebi.ac.uk/metabolights/MTBLS123

These “other” scaling methods include level scaling, range scaling, VAST scaling, Pareto scaling, and autoscaling (Ebbels et al. 2011; Craig et al. 2006). In Fig. 6, we show the effects of several scaling and transformation methods on urine metabolite concentration data. Each scaling method had its own strengths and weaknesses. For example, autoscaling can often increase noise artefacts from spectral regions devoid of usable signals. To address this problem, Pareto scaling uses the square root of the standard deviation instead of the standard deviation as the scaling factor. This increases the sensitivity and reduces noise, while still allowing the data to remain closer to the original measurements (Ebbels et al. 2011). Variable stability scaling (VAST) is another method that weighs each variable according to its measured stability and then down-weights the variables that are less stable. This approach is believed to improve the distinction between different classes in subsequent multivariate analysis (Keun et al. 2003). The advantages of this method were first demonstrated by analysing NMR spectra of urine in an animal model of bilateral nephrectomy (Keun et al. 2003).

Numerical transformations (e.g. power or logarithmic transformation) are another example of scaling or statistical normalization. Transformations are mostly used to correct for *heteroscedasticty* or to correct for data skewness and non-normality before statistical testing. When power and log transformations (or more sophisticated transformation like the Box–Cox’s transformation) are used, large values are more heavily penalized than small values. This provides a pseudo-scaling effect that can be particularly relevant to NMR data as it enhances the importance of small peaks relative to larger ones (Sakia 1992; Kvalheim et al. 1994). Although working on a different context, Feng et al. cautioned against the use of logarithmic transformation noting that the results of standard statistical tests performed on log-transformed data are often not relevant for the original, non-transformed data (Changyong 2014).

The optimal transformation method should be capable of reducing or removing heteroscedastic noise (i.e. variables of sub-group are different than other sub-groups) into homoscedastic information (i.e. variables are similar in sub-groups). These methods are more relevant when reducing non-linear, non-additive, non-normalized or heteroscedastic noise in NMR data and will enhance the information contained in small peaks (Sakia 1992; Kvalheim et al. 1994). For instance, the Box–Cox transformation is a parametric power transformation method used for nonlinear conversion of data where large values are reduced relatively more than the small values (Ebbels et al. 2011; Sakia 1992).

Van den Berg et al. reviewed most of the methods presented in Table 5 using MS data and found that auto-scaling and range scaling performed better with regard to biological interpretation when data were analysed using PCA. In particular, these two methods were able to remove the dependence of metabolite rank importance in the PCA model from the average concentrations and the magnitude of fold changes. They also found that centering, log transformations, and power transformations, along with level and Pareto scaling showed a strong dependence on concentration and fold changes leading to poorly interpretable PCA results. However, Kohl et al. (2012) found VSN to outperform the latter two methods in a more exploratory setting.

In many situations, high concentration and high variance metabolites may not be the most relevant to the biological problem being studied. However, since most (multivariate) statistical approaches use the information embedded in the variance/covariance matrix, it is crucial that the variance structure of the data is preserved because it contains valuable (biological) information. However, the choice of the scaling methods needs to be tailored on both the application and the data type. NMR and MS data have inherently different properties in term of range and error structure and this may explain the different performance of the same method when applied on different data from different platforms. Depending on the final application, for NMR binned data, Pareto scaling may be the most sensible choice when the aim is data exploration through PCA. In a more discriminant setting, Parsons et al. (2007) found generalised logarithm transformations to significantly improve the discrimination between sample classes yielding higher classification accuracies compared to unscaled, auto-scaled, or Pareto scaled data (Parsons et al. 2007).

Gromski et al. (2015) investigated the effect of autoscaling, range scaling, level scaling, Pareto scaling and VAST scaling on four classification models [principal components-discriminant function analysis (PC-DFA), support vector machines (SVM), random forests (RF) and *k*-nearest neighbours (kNN)] and found that VAST scaling was more stable and robust across all the classifiers considered and advocated for its use.

Our recommendation is that scaling and transformation should be done on all NMR-derived biofluid data prior to conducting multivariate statistical analyses. Visualization and assessment of the scaling and/or transformation effects on the data is necessary to ensure that these scaling or transformation efforts make the data more centred and more Gaussian in its overall distribution (i.e. reducing heteroscedasticity). Researchers must refrain from blindly (i.e. without visualizing the consequences) applying different transformation and scaling methods until the results of the analysis match some predefined hypothesis, as this is scientifically and statistically improper.

### Multivariate statistics, compound identification and biological interpretation

Once all the NMR data has been properly prepared through the careful use of phasing, weighting functions (apodization), zero filling, baseline correction, normalization, and scaling (among other methods described previously and in the referenced materials), then the specialized work of statistical analysis, compound identification and biological interpretation may begin. There are many excellent reviews on how to conduct multivariate statistics with MS or NMR-based metabolomics data (Ren et al. 2015; Emwas et al. 2013; Izquierdo-Garcia et al. 2011) as well as on methods to perform compound identification and biological interpretation from NMR data (Karaman et al. 2016; Donaa et al. 2016; Schleif et al. 2011). It is well beyond the scope of this paper to provide an overview or an assessment of these subjects. However, a few comments or suggestions are perhaps worthwhile.

In the field of NMR-based metabolomics there are a number of well-regarded, freely available software tools and resources that are widely used and which we highly recommend. These include: MetaboAnalyst (Xia et al. 2009, 2015; Xia and Wishart 2010) for multivariate analysis, metabolite annotation and biological interpretation, MVAPACK for multivariate analysis (Worley and Powers 2014), Workflow4Metabolomics for multivariate analysis (Giacomoni et al. 2015), Metassimulo for multivariate analysis (Muncey et al. 2010), the Human Metabolome Database (HMDB) for metabolite annotation and biological interpretation (Wishart et al. 2013), and the BioMagResBank (BMRB) for metabolite identification (Markley et al. 2008). There are also a number of commercial tools such as Chenomx’s NMR Suite, Bruker’s AMIX software, MestreLab’s MNova and Umetrics SIMCA that offer tools for multivariate analysis and/or metabolite identification. While many researchers prefer to do their own statistical analysis and data interpretation, our recommendation is, for those who are new to metabolomics, that they should collaborate with an individual who has already had significant prior experience in metabolomic data analysis and data interpretation. Alternately, statistical neophytes should dedicate considerable time and effort to become a proficient in this area as possible, prior to embarking on this sort of analysis.

## Conclusion

The intent of this review was to provide readers with some guidance and recommendations regarding how to process and post-process NMR spectral data collected on biofluids, with a particular focus on urine. The wide disparity in published practices and outcomes from different NMR metabolomics laboratories led us to investigate existing practices and to systematically assess which methods worked best under which situations. In doing so, we have tried to highlight the advantages and disadvantages of different NMR spectral collection and spectral data processing steps that are common to NMR-based metabolomic studies of biofluids such as urine. More specifically we reviewed the existing literature, assessed the methods in our laboratories and made the following best-practice recommendations:

1. We recommend the use of DSS (especially deuterated DSS) as the chemical shift reference standard for all urinary NMR spectroscopy.
2. We recommend that auto-phasing should be used as an initial phasing step. Subsequently, all biofluid NMR spectra should be manually inspected for phase distortions and, if necessary, those spectra exhibiting phase distortions should be phased manually.
3. We recommend that all biofluid NMR spectra should be manually inspected for baseline distortions and, if necessary, those spectra exhibiting baseline distortions should be corrected using specific high quality baseline correction software (mentioned in this document).
4. For urine NMR spectra we recommend the removal of the upfield region (0.00–0.60 ppm), the residual water region (~ 4.50–4.9 ppm) and the urea region (5.5–6.1 ppm), especially prior to alignment and binning.
5. We recommend that icoshift should be used in the alignment of biofluid (esp. urine) NMR spectra.
6. No specific recommendation on the best spectral binning method is possible, although equidistant binning appears to be the simplest and fastest approach.
7. When possible, we recommend physiological normalization for NMR-based urinary metabolomic studies, with specific gravity being preferred over creatinine normalization. In situations where physiological normalization is not possible, we recommend Quantile normalization for large (> 50 samples) data sets while Spline normalization is recommended for smaller data sets.
8. We recommend that scaling and transformation should be done on all NMR-derived biofluid data prior to conducting multivariate statistical analyses and subsequent compound identification or biological interpretation. Furthermore, this scaling and transformation must be visualized and assessed by users to determine if the heteroscedasticity has been properly reduced.

Following these recommendations should allow users not only to get consistent, reproducible NMR data but also to optimize the outcome for their multivariate statistical analysis as well as their subsequent final data interpretation.

This review is not intended to be prescriptive. Describing a single protocol that works for all situations is simply not practical. Indeed, the optimal choice of data processing (and post-processing) options depends on the experiment being conducted, the quality of the data at hand, along with an appreciation of the problem being addressed. For example, if the focus of a study is on exploring differences between groups or subgroups, one should always try to employ a normalization and scaling strategy that will not level out possible differences. If the focus in on data exploration, it is advisable to scale the data in such a way as to avoid using high variance values that will dominate the final model. In all cases, careful experimental preparation prior to any NMR data acquisition, followed by careful, consistent spectral processing and post-processing is necessary before a truly productive NMR data analysis can begin. Otherwise precious time and resources will be wasted on trying to interpret inconsistent data and inaccurate results.
